# Microspherical Particles of Solid Dispersion of Polyvinylpyrrolidone K29-32 for Inhalation Administration

**DOI:** 10.1155/2018/2412156

**Published:** 2018-01-10

**Authors:** L. S. Usmanova, M. A. Ziganshin, I. T. Rakipov, N. M. Lyadov, A. E. Klimovitskii, T. A. Mukhametzyanov, A. V. Gerasimov

**Affiliations:** ^1^Department of Physical Chemistry, A.M. Butlerov Institute of Chemistry, Kazan Federal University, Kremlevskaya 18, Kazan 420008, Russia; ^2^ZPTI-Subdivision, FIC KazanSC of RAS, Sibirsky Tract 10/7, Kazan 420029, Russia; ^3^Institute of Engineering, Kazan Federal University, Kremlevskaya 18, Kazan 420008, Russia

## Abstract

Inhalation administration is a promising alternative to the invasive drug delivery methods. The particle size required for ideal drug aerosol preparation is between 1 and 3 *μ*m. The application of microspherical particles of solid dispersions enhances bioavailability of poorly soluble drugs due to the solubilization. In the present work, the spray drying process of the production of microspherical particles of solid dispersions of polyvinylpyrrolidone K29-32 with model hydrophobic drug, phenacetin, was optimized using the results of DSC, PXRD, and viscometry. The diameter of the obtained particles is within 1–3 *μ*m range. The Gibbs energy of dissolution in water was shown to be negative for the mixture with polymer/phenacetin mass ratio 5 : 1. We have demonstrated that the optimal size distribution for the inhalation administration is obtained for microspherical particles produced using spray caps with 7.0 *μ*m hole size. The dissolution rates of phenacetin from the produced microspherical particles were faster than that of drug powder. As evidenced by powder X-ray diffraction data, phenacetin stayed in amorphous state for 4 months in microspherical particles of solid dispersions. According to the obtained results, strategic application of the spray drying process could be beneficial for the improvement of the pharmaceutical properties of model drug, phenacetin.

## 1. Introduction

Inhalation therapy is an express method of drug delivery into the human circulatory system. Due to extremely abundant capillary network and enormous alveoli area drugs are absorbed extremely rapidly [1, 2]. The inhaled chemicals are not subjected to the biotransformations, which occur in the digestive tract, and drug activity is not reduced in the liver [3]. Drugs introduced into lungs via inhalation have 10 to 200 times higher bioavailability than by nasal or gastrointestinal administration [4]. Furthermore, inhalation administration of drug compound in an aerosol with controlled particle size and shape may serve as a noninvasive alternative to injection of medicines.

In an inhalation therapy, inhaled drugs enter the body as aerosols. Therapeutically active particle size is limited between 1 and 5 *μ*m [5]. Particles with a diameter higher than 10 *μ*m are deposited in the oropharynx, those measuring between 5 and 10 *μ*m accumulate in the central airways, and those from 0.5 to 5 *μ*m enter the small airways and alveoli [6]. In order to target the alveolar region specifically, the aerosol particle diameter should not exceed 3 *μ*m [7]. Particles with a diameter below 1 *μ*m are exhaled during normal tidal breathing [7]. Moreover, the work [8] demonstrated that particles less than 1 *μ*m in size are more readily subjected to phagocytosis. Therefore, the ideal drug aerosol must have particles with a diameter in 1 to 3 *μ*m range [2].

On the other hand, most novel drugs are poorly soluble in water limiting their bioavailability [9, 10]. The most convenient means for “hydrophilization” of drugs is solid dispersion formation [11, 12].

Biocompatible polymers including polyvinylpyrrolidone (PVP) have found wide application as water-soluble matrix for drug delivery [13, 14].

It was earlier demonstrated that PVP produces solid dispersions with a wide range of compounds [15–20] and that a decrease of cytotoxicity of active pharmaceutical ingredients (API) occurs due to solid dispersion formation [21]. The fact that PVP shows protective properties towards macrophage action on compounds encapsulated in the polymer is shown [22, 23].

It must be considered that polymers used for solubility increase not only affect pharmacological properties of drugs but also have their own activity profile and toxicity [7, 24, 25]. In this respect, PVP is among most promising materials as its biocompatibility and its usage as plasma replacement illustrate the almost complete lack of toxicity [26–28].

The main problem of solid dispersion production is the determination of the range of polymer : drug composition in which crystalline drug is still not found while the composite is readily soluble in water.

Amorphous glassy state should provide stability towards degrading action of oxygen and humidity due to high viscosity and density. The glass transition temperature is an important parameter controlling the stability of an amorphous state. Therefore, the end product of solid dispersion formation (including spray drying) should has the glass transition temperature significantly higher than room temperature. It must be noted that pharmaceutical substance due to strong intermolecular interactions with polymer matrix may not only act as a plasticizer [29] but also affect the rate of release [30], which further complicates optimization of the polymer-drug ratio.

Presently one of the main methods of nano- and microparticle preparation is the spray drying process, which has found a good use for commercial-scale production for inhaler products. This method enables the production of spherical particles with sharp size distribution [31], as well as solid dispersions of drugs [32]. Furthermore spherical particles demonstrate lower toxic effects than that of other shapes [33], which makes spray drying the most promising method for the preparation of medical products for inhalation administration. Only a limited number of commercially available devices allow production of spherical particles of the 1–3 *μ*m size range. The production rate of such devices is usually limited and strongly depends on the viscosity (concentration) of the feeder solution and temperature (feed rate) of the desiccant [34, 35]. The parameters of the spray drying process also have a significant effect on the morphology of produced particles [36–40].

For example, it is noted [41] that the use of viscous polymer solutions in the spray drying process decreases the yield of target product by 0.5–10%. Furthermore, high hygroscopicity of solution may lead to the formation of particles with broad size distribution due to aggregation during the spray drying process and after that [42]. Particles with broad size distribution cannot be used for inhalation delivery.

Thus the development of the production method of the micrometer-size spherical solid dispersion particles based on polyvinylpyrrolidone via spray drying process as well as optimization of such process is an important current problem of the modern pharmacology.

In the present work, a composition of solid dispersions of polyvinylpyrrolidone K29-32 with well-established model hydrophobic drug compound, phenacetin [43–45], was optimized based on data from a combination of physicochemical methods. The optimal concentration of drug components in solution was found which enables the production of spherical microparticles with a mean diameter in the range between 1 and 3 *μ*m, suitable for inhalation administration and allowing relatively high dissolution rate. Preliminary minimal set of physicochemical methods required to produce PVP solid dispersions using spray drying process includes (but is not limited to) the following: differential scanning calorimetry and X-ray powder diffraction as well as determination of rheological properties of the solution and quantitative dissolution process investigation.

## 2. Materials and Methods

### 2.1. Materials

Polyvinylpyrrolidone K29-32 MW 58,000 (PVP K29-32) (Acros Organics, Lot A0357351) and phenacetin, 98% (PHE) (Aldrich, Lot #BCBD7322V), were used as received. Absolute ethanol and bidistilled water were used as solvents.

### 2.2. Preparation of Physical Mixture

Mechanical mixtures of PVP K29-32 with PHE were prepared by mixing measured quantities of substances in an agate mortar until complete homogenization was achieved. Mass ratios of PVP K29-32 : PHE were 1 : 1, 2 : 1, 3 : 1, 4 : 1, 5 : 1, 6 : 1, 7 : 1, 8 : 1, 9 : 1, and 10 : 1. The resulting mixture was a white powder.

Composites of PHE/polymer were prepared by melting of mechanical mixtures with the required content of components in the ratios 1–10 : 1 by weight. Melting was carried out under an inert atmosphere at 135°C with subsequent cooling down to room temperature.

### 2.3. Simultaneous Thermogravimetry, Differential Scanning Calorimetry, and Mass-Spectroscopy (TG/DSC/MS)

Simultaneous thermogravimetry and differential scanning calorimetry (TG/DSC) analysis of solid samples and mass spectrometric (MS) evolved gas analysis were performed using the thermoanalyzer STA 449F1 Jupiter (Netzsch, Germany) coupled with quadrupolar mass-spectrometer QMS 403 D Aeolos (Netzsch, Germany) at the temperature range of 40–200°C (40–500°C for initial samples). In each experiment, the temperature scanning rate was 5°C/min, and an argon atmosphere with a total flow rate of 75 ml/min was used [46].

### 2.4. Differential Scanning Calorimetry (DSC)

Enthalpies and transition temperatures of PHE and PVP K29-32, as well as their mechanical mixtures in the temperature range of −60–160°C (−60–200°C for PVP K29-32), were determined using differential scanning calorimeter DSC 204 F1 Phoenix (Netzsch, Germany), as described earlier [47, 48]. Measurements were carried out on samples weighing 5.5–12.1 mg, at a heating rate of 5°C/min (cooling 10°C/min), in a dynamic atmosphere of argon (150 ml/min).

### 2.5. Powder X-Ray Diffraction

Powder X-ray diffraction (PXRD) studies of polymer, PHE, and their composites were made using a MiniFlex 600 diffractometer (Rigaku, Japan) equipped with a D/teX Ultra detector. In this experiment, Cu K*α* radiation (40 kV, 15 mA) was used and data were collected at room temperature in the range of 2*θ* from 3 to 50° with a step of 0.02° and exposure time at each point of 0.24 s without sample rotation [46, 49].

### 2.6. Limiting Solubility of Phenacetin

UV-spectroscopy (Cary 100 UV-Vis Spectrophotometer (Agilent Technologies, Germany)) was used to determine the effect of PVP K29-32 on limiting solubility of PHE. A series of solutions was prepared with a fixed content of drug 20 mg/ml at different PVP K29-32 : PHE ratios (1 : 1, 2 : 1, 4 : 1, 6 : 1, 8 : 1, and 10 : 1). After 24 hours, solutions were filtered out from the undissolved part of PHE using a filter of 0.22 *μ*m pore diameter. The increase of PHE content in water at 25°С was determined as a ratio of the optical density values at 245 nm (with suitable dilutions), obtained in the presence of a different quantity of polymer and without it.

Gibbs free energy value (Δ_*S*_*G*), related to the process of dissolution of PHE in an aqueous solution of PVP K29-32, was calculated for each concentration of polymer as described previously [50].

### 2.7. Study of the Viscosity Properties

For optimization of spray drying process, rheological properties of PVP K29-32 ethanol solutions were studied. Solutions with PVP K29-32 content of 0.05, 0.1, 0.5, 1.0, 2.5, 3.5, and 5.0% w/v were prepared to perform the measurements. Viscosity and density assays were carried out using velocity meter DSA 5000 (Anton Paar, Austria) at 20°C, 25°C, 30°C, 35°C, 37°C, and 40°C [51]. The temperature was controlled by a built-in Peltier thermostat within ±0.002°C.

### 2.8. Spray Drying

PVP K29-32/PHE solution (2.5% w/v) was prepared by dissolving 0.8334 g of PVP K29-32 powder and 0.1666 g of PHE in 40 ml of ethanol. Solid dispersion particles were prepared by Nano Spray dryer B90 (Buchi, Switzerland) operated in the open mode with three different spray caps (4.0, 5.5, and 7.0 *μ*m hole size): flow rate was 7 ml/min; drying air flow 130 l/min; and inlet temperature 65°C. Dried solid dispersion particles were collected from the particle collecting chamber using a scraper, and the collected powders were kept in Eppendorf type tubes at room temperature (RT).

### 2.9. Fourier Transformation-Infrared Spectroscopy (FTIR) Analysis of Solid Samples

FTIR spectra (600–4000 cm^−1^) were acquired for solid samples of pure PVP K29-32, PHE, and their composites. Data were collected using a Vertex 70 FTIR spectrometer (Bruker, Germany) with a single reflection and germanium crystal ATR accessory (MIRacle, PIKE Technologies, USA) purged under dry air to remove atmospheric water vapor. Background spectra of 128 scans at a resolution of 2 cm^−1^ were subtracted from the sample spectra [50].

### 2.10. Morphologies of Microparticles

Sample morphology studies were performed on Zeiss EVO 50 XVP scanning electron microscope (Carl Zeiss, Germany) in variable pressure mode. This mode is suitable for nonconducting and gas-emitting samples. In the present study, the pressure in the working chamber was 20–30 Pa. For image acquisition, tetra-solid-state BSE detector (reflected electron detector) was used. Samples were visualized using an acceleration voltage of 20.0 kV. Image-Pro Plus 6.0.0.260 and Origin 8.1 software packages were used to build distribution curves.

### 2.11. Drug Contents in Microparticles

**Figure 1:**
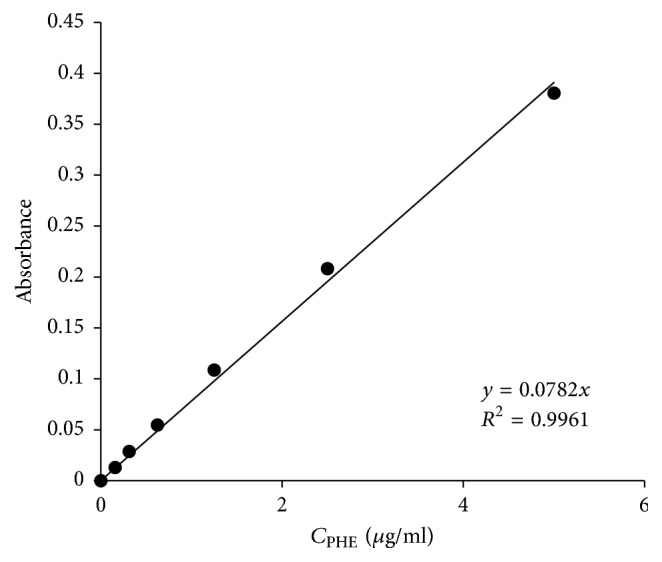
Calibration curve for phenacetin concentration in ethanol. Absorbance values are corrected for dilution.

The total drug content in the spray-dried particles was assayed by dissolving the solid dispersion in ethanol as described in [52]. The concentration of microparticles in ethanol was 10 mg/ml. The mixture was stirred continuously overnight at RT. Solutions were then analyzed with Cary 100 UV-Vis Spectrophotometer (Agilent Technologies, Germany) at 245 nm with suitable dilutions. The drug content was calculated using regression equation (Figure 1).

### 2.12. *In Vitro* Dissolution

PHE dissolution kinetics from composites was assayed with Dissotest CE1 (Sotax, Switzerland) (USP IV) in a closed loop [53]. Samples containing an equivalent amount of PHE (12.5 mg) were used for dissolution investigation.

The test was performed at 37.0°C with 17 ml/min flow rate, employing phosphate buffer with pH = 6.86 as dissolution medium. At different times (after 1, 2, 3, 4, 5, 10, 15, 20, 25, 30, 60, 90, 120, 150, and 180 minutes) a set solution volume was taken for analysis. Taken volume was replaced with the same amount (4 ml) of fresh medium to replenish solution. PHE content was measured spectrophotometrically using Cary 100 UV-Vis Spectrophotometer (Agilent Technologies, Germany) at 245 nm with suitable dilutions.

### 2.13. Statistical Analysis

Statistical analyses were performed using Student's *t*-test or one-way analysis of variance (ANOVA). A *P* value of <0.05 was considered significant for all analyses. All data are expressed as a mean and standard deviation from at least three independent experiments.

## 3. 3. Results and Discussion

### 3.1. Results of Thermoanalysis of Individual Compounds and Their Physical Mixtures

**Figure 2:**
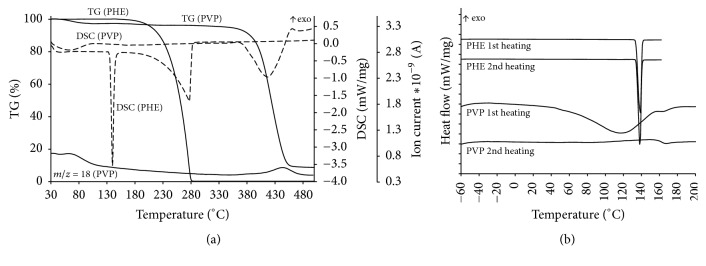
(a) Results of TG/DSC/MS analysis of PHE and PVP K29-32 in the dynamic argon atmosphere 75 ml/min in the temperature range 40–500°С. Ion thermogram of H_2_O (*m*/*z* = 18) is shown. Heating rate is 5°С/min. (b) DSC curves of the initial samples of PVP K29-32 and PHE in the dynamic atmosphere of argon 150 ml/min. Heating rate is 5°C/min.

The results of combined TG/DSC/MS analysis of PHE and PVP K29-32 are presented in Figure 2(a). There is no observed mass loss for PHE in the temperature range 40–160°С.

A thermal decomposition of PHE is observed above 180°С; therefore low-temperature DSC measurements of composites were limited to the temperatures below 160°С. A distinct endoeffect occurs between 130 and 175°С on the DSC curves, which corresponds to drug melting. Thermal effects on the DSC curve above 180°С are related to thermal decomposition of the drug. A mass loss (3.4%) combined with endoeffect within 40–200°С is observed for polyvinylpyrrolidone K29-32; both are related to the elimination of water (*m*/*z* = 18). Decomposition of PVP K29-32 is observed above 350°С.

Low-temperature differential scanning calorimetry was employed for more accurate analysis of thermal effects of phase transitions including low-temperature region. The results of DSC analysis of PHE and PVP K29-32 are presented in Figure 2(b).

Endoeffects of melting with onsets at 134.4°С and 134.1°С and enthalpies 188.8 J/g and 188.7 J/g for first and second heating, respectively, are observed on the heating curves of PHE (Figure 2(b)). Temperature and enthalpy values agree with previous measurements [45]. No additional effects in the studies temperature range are observed on DSC curves. An endoeffect related to the elimination of water is visible on the first heating DSC curve of polyvinylpyrrolidone K29-32 and a glass transition afterward. A glass transition at *T*_*g*_ = 163.0°С is more clear on the second heating.

**Figure 3:**
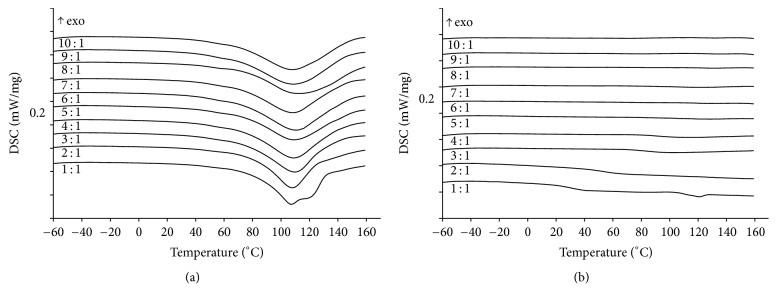
DSC curves of PVP K29-32/PHE mechanical mixtures of different compositions (1–10 : 1) in dynamic argon atmosphere 150 ml/min and temperature range −60–160°С. (a) First heating; (b) second heating. Heating rate is 5°С/min.

Thermophysical properties of mechanical mixtures of PVP K29-32 with PHE with 1 : 1, 2 : 1, 3 : 1, 4 : 1, 5 : 1, 6 : 1, 7 : 1, 8 : 1, 9 : 1, and 10 : 1 compositions were studied using low-temperature DSC in the temperature range −60–160°С. Analysis results are presented in Figure 3.

On the first heating of physical mixtures of PVP K29-32 and PHE (Figure 3(a)) endoeffects related to the elimination of water are observed. The water content in the studied samples, determined using TG/DSC/MS analysis, is between 3.0 and 4.8%. No other effects are observed for PVP K29-32 : PHE 2–10 : 1 mixtures. In the same time, a shoulder of the endoeffect of PHE melting is found for the 1 : 1 mixture. This fact supports the lack of crystal phase of PHE in composites with compositions 2–10 : 1.

On the second heating of studied PVP K29-32 : PHE mixtures (Figure 3(b)) thermal effect of PHE melting is also observed in 1 : 1 mixture. Melting of PHE is not visible in other mixtures. Furthermore, for mixtures of 1–5 : 1 composition a glass transition of PVP K29-32 is clearly visible with *T*_*g*_ 33.0°С (1 : 1), 55.0°С (2 : 1), 84.0°С (3 : 1), 89.7°С (4 : 1), and 98.5°С (5 : 1). Such behavior evidences plasticizing effect of PHE and must be considered for the optimization of spray drying process as the temperature of drying must be at least 10°С below *T*_*g*_ [54].

Therefore based on DSC data the optimal composition of PVP K29-32 : PHE mixture is 5 : 1, because for this mixture no separate crystal phase of PHE was observed, glass transition of PVP K29-32 has a temperature well above spray drying process temperature, and the content of drug is maximal.

### 3.2. PXRD of Initial Compounds and Their Physical Mixtures

**Figure 4:**
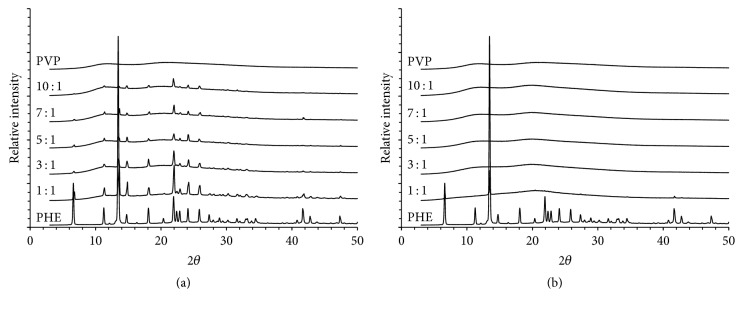
Powder X-ray diffractograms of PVP K29-32, PHE, their physical mixtures of different compositions (a), and same mixtures after melting (b).

Figure 4 demonstrates the results of powder X-ray diffractometry of the initial samples of polyvinylpyrrolidone K29-32, PHE, and their physical mixtures (Figure 4(a)) of 1 : 1, 3 : 1, 5 : 1, 7 : 1, and 10 : 1 composition (by weight) as well as same mixtures after melting (Figure 4(b)).

Reflections characteristic for PHE are visible in all diffractograms of physical mixtures. On the diffractograms of samples after melting, peaks of PHE are only observed in mixture with 1 : 1 PVP K29-32 : PHE composition. A halo common for the amorphous phase is present in diffractograms for all other compositions, which correspond to the formation of solid dispersions. It must be noted that results of DSC and PXRD agree with each other. Therefore a combination of two different methods allows making a definite choice of optimal composition of PVP K29-32 and PHE for the formation of solid dispersion using spray drying process; this composition is 5 : 1 by weight.

### 3.3. Thermodynamics of Phenacetin Dissolution

Limiting solubility of PHE in water in presence of polyvinylpyrrolidone K29-32 was studied using UV-spectrophotometry.

**Figure 5:**
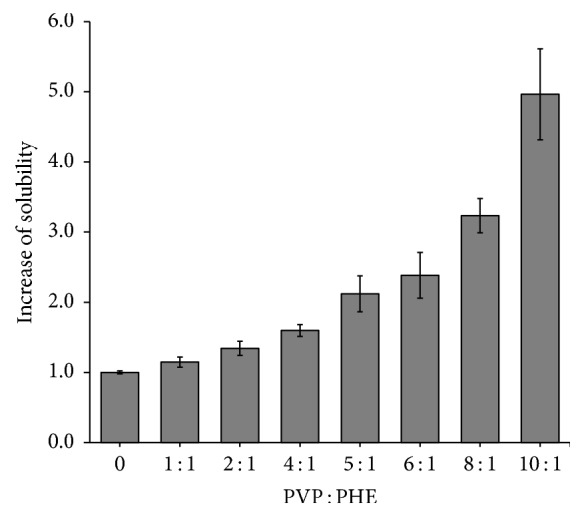
Results of UV spectrophotometric analysis of aqueous solutions of mixtures of PVP K29-32 and PHE at different component compositions. The optical density is measured at a wavelength of 245 nm.

As visible from Figure 5 PHE concentration in solution increases upon the increase of polymer content. Compared to the solution of PHE without PVP K29-32 maximum solubility increase is 4.9-fold.

The results obtained for Gibbs free energy of dissolution (Table 1) demonstrate the spontaneity of the solubilization process for polymer-drug compositions (5–10) : 1. In general, the increase in solubility is directly associated with values of Δ_*S*_*G* < 0, which is usually proportional to the increase in concentration of polymer, in such a way that the more negative the value of Δ_*S*_*G*, the better the solubilizing effect [17].

Therefore solution thermodynamics data for PHE in the presence of polyvinylpyrrolidone K29-32 are not contradicting previously determined optimal PVP K29-32 : PHE composition of 5 : 1. Solubility increase for this composition is 2 times the value of pure PHE.

### 3.4. Viscosity Study of PVP K29-32 Solution

Solution viscosity is one of the critical factors for a spray drying process and, together with surface tension, defines the size of the droplets formed [55, 56]. Therefore, a study of the kinematic and dynamic viscosities of PVP K29-32 solutions in ethanol depending on polymer concentration was carried out.

**Figure 6:**
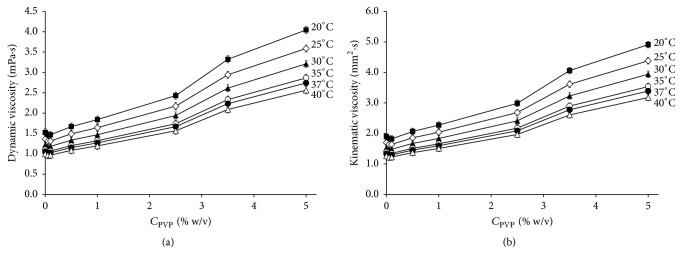
Dependence of dynamic (a) and kinematic (b) viscosity on PVP K29-32 concentration in ethanol at different temperatures.

As evident from Figure 6 increase of PVP K29-32 concentration up to 2.5% w/v causes an almost linear increase of solution viscosity at all temperatures studied. The magnitude of solubility increase as compared to pure ethanol is not higher than 1.6-fold. Further PVP K29-32 content increase in ethanol up to 3.5% w/v leads to a relatively sharp viscosity increase. Similar behavior was previously shown for PVP K29-32 solutions in water and alcohols [57, 58]. Because of this for a spray drying process, a concentration of PVP K29-32, 2.5% w/v, was chosen.

### 3.5. Physicochemical Properties of Phenacetin Spray-Dried Solid Dispersion

Average product yields of spray drying were 47 ± 2, 60 ± 2, and 61 ± 3% for particles, formed with 4.0, 5.5, and 7.0 *μ*m hole size, respectively. Dried samples had the appearance of white powders and were analyzed using several physicochemical methods. Moreover, in a separate independent experiment, it was demonstrated that spray drying does not commence at PVP K29-32 concentration over 3.5% in solution, because of high solution viscosity. This supports the choice of solution concentration made from viscosity measurements.

#### 3.5.1. Solvent Content in Microparticles

**Figure 7:**
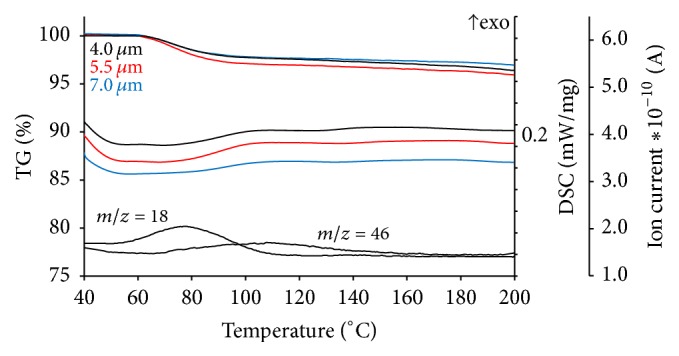
TG/DSC/MS results of microspherical particles formed by spray drying with three different spray caps (4.0, 5.5, and 7.0 *μ*m hole size) in dynamic argon atmosphere 75 ml/min. Heating rate is 5°С/min. Ion thermograms *m*/*z* = 18 and *m*/*z* = 46 correspond to water and ethanol, respectively (data is shown for particles formed by spray drying with spray caps 5.5 *μ*m hole size).

Residual solvent content in microspherical particles was investigated using TG/DSC/MS. The results are presented in Figure 7. For all studied samples, the mass loss between 40 and 140°С is below 3.6%. This loss of mass is related to water and ethanol elimination as evidenced by the ion thermograms for *m*/*z* = 18 and *m*/*z* = 46, respectively. The solvent loss is accompanied by the endoeffect on DSC curves. The majority of water is lost below 100°С, which supports the lack of strong intermolecular interactions and is a prerequisite for good storage stability including high humidity conditions.

A presence of water in produced solid dispersions may be attributed to the hygroscopicity of the PVP polymers [59].

It must be noted that water constitutes the majority of the mass loss of samples, while ethanol is eliminated at temperatures above its boiling point; this points to the formation of relatively strong solvate complexes during spray drying. However, the amount of ethanol bond is negligibly low.

Therefore, it can be concluded that spray drying parameters (inlet temperature and drying air flow) allow the formation of microparticles with relatively low residual solvent content.

#### 3.5.2. Differential Scanning Calorimetry

**Figure 8:**
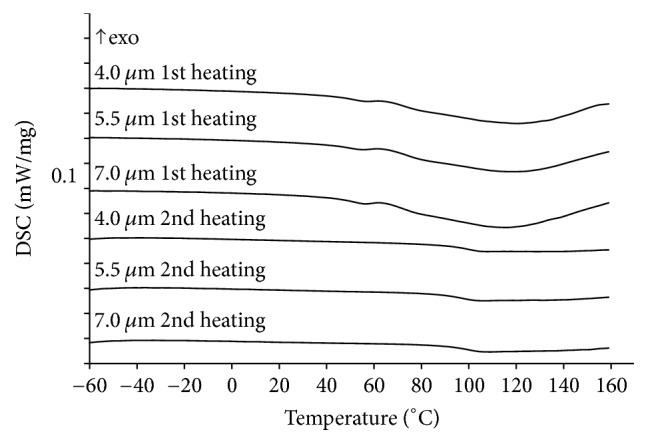
DSC curves of microspherical particles formed using spray drying process with three different spray caps (4.0, 5.5, and 7.0 *μ*m hole size) in dynamic argon atmosphere 150 ml/min. Heating rate is 5°С/min.

Upon the first heating of microparticles endoeffects related to the loss of solvents are visible on DSC curves (Figure 8). No other effects are found. Polyvinylpyrrolidone K29-32 glass transitions at 99.7, 99.6, and 97.2°С for particles formed using 4.0, 5.5, and 7.0 *μ*m hole size, respectively, are present on DSC curves of second heating. This temperature is close to the glass transition observed upon second heating in a physical mixture PVP K29-32 : PHE 5 : 1 (98.5°С) (Figure 3(b)). This means that the drug content in microparticles is close to theoretical values.

Relatively high glass transition temperature promotes good storage stability at room temperature; the prolonged physical stability of amorphous materials is possible if storage temperature is at least 50°C below the glass transition [60].

A lack of endoeffects of PHE melting is an indirect evidence of the formation of solid dispersion during spray drying.

#### 3.5.3. PXRD of Microparticles

**Figure 9:**
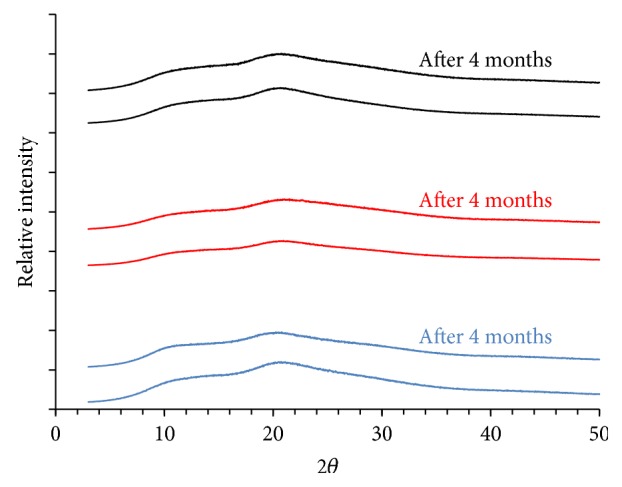
Powder X-ray diffractograms of freshly generated microspherical particles prepared with three different spray caps (4.0 *μ*m (black), 5.5 *μ*m (red), and 7.0 *μ*m (blue) hole size) and after 4-month storage in ambient conditions.

No characteristic reflections of PHE are observed on powder diffractograms (Figure 9), which supports the lack of crystal phase in the particles and further supports earlier conclusion on the formation of solid dispersions during spray drying.

No crystal phase of PHE was found upon 4-month storage in ambient condition (Figure 9).

#### 3.5.4. FTIR Spectroscopy of Microparticles

**Figure 10:**
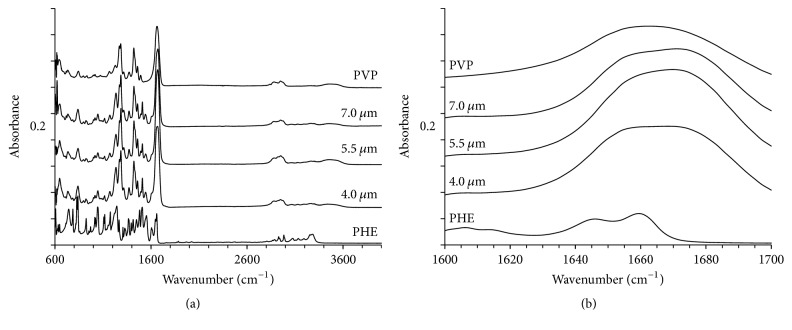
IR spectra of PVP K29-32, PHE, and microspherical particles formed using spray drying with three different spray caps (4.0, 5.5, and 7.0 *μ*m hole size) in the wavenumber range 600–4000 cm^−1^ (a) and 1600–1700 cm^−1^ (b).

IR spectra of PHE and polyvinylpyrrolidone K29-32 powders, as well as microspherical particles produced by spray drying process with three different spray caps, are demonstrated in Figure 10. Spectra of all microspherical particles are visibly similar (Figure 10(a)), which supports the closeness of the prepared solid dispersions. PHE absorbance bands at 1646 and 1659 cm^−1^ correspond to free and H-bonded C=O groups in crystals [50].

A shoulder of the band related to the free C=O PHE group is visible on the IR spectra of microspherical particles (Figure 10(b)), while no bonded carbonyl group signal (1646 cm^−1^) is present. This observation further supports the lack of PHE crystal phase growth during the spray drying process and solid dispersion formation with PVP K29-32, as in the latter PHE is present in amorphous condition and prevented from the H-bonding of the PHE-PHE type. Furthermore, a capacity of PVP K29-32 towards H-bonding with PHE can effectively prevent the crystallization [61, 62]. The absence of chemical changes of the drug after spray drying is supported by the lack of changes in the position of the characteristic IR-bands of phenacetin between the initial substance and resultant microparticles [30].

#### 3.5.5. Shape and Size Distribution of Microparticles

**Figure 11:**
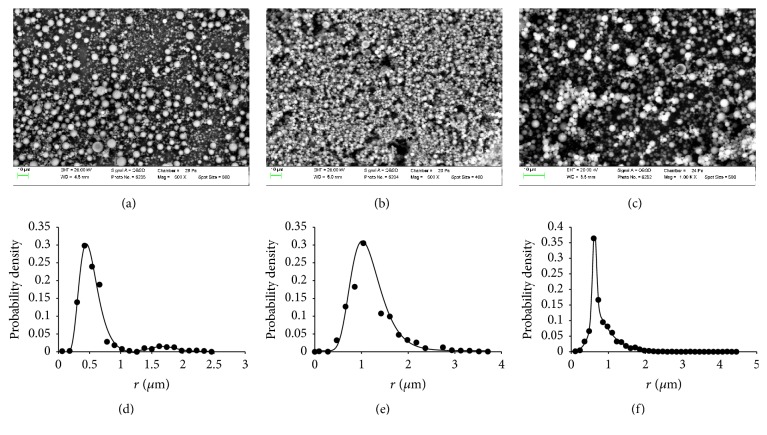
SEM images of PVP K29-32/PHE composites, formed using spray drying process with three different spray caps with 4.0 *μ*m (a), 5.5 *μ*m (b), and 7.0 *μ*m (c) hole size and size distribution curves calculated from these images for spray caps with 4.0 *μ*m (d), 5.5 *μ*m (e), and 7.0 *μ*m (f) hole size.

SEM images of microspherical particles formed using spray drying process with three different spray caps together with size distribution curves are presented in Figure 11.

Prepared particles have a spherical shape (Figures 11(a)–11(c)). Formation of spherical particles with no surface defects corresponds to a lack of external shell growth during the spray drying because of low concentration [40]. A lack of polymer shell also reflects the homogeneity of the solid dispersion and good affinity of PHE towards polyvinylpyrrolidone K29-32. It must be noted that a regular spherical shape together with the lack of agglomeration should promote good particle aerodynamics.

Distribution curves were plotted based on the data from 3801, 4238, and 2193 particles and were described using amplitude version of Gaussian peak function, probability density function of random variable whose logarithm is normally distributed, and Chesler-Cram peak function for spray caps with 4.0 *μ*m, 5.5 *μ*m, and 7.0 *μ*m hole size, respectively (Figures 11(d)–11(f)).

Two maxima are present on size distribution curve of spray caps with 4.0 *μ*m hole size. This can be explained by the formation of bigger particles via agglomeration of droplets at the time of their generation, which reduces surface energy. A single peak is found on the size distribution curves of particles formed with spray caps of 5.5 *μ*m and 7.0 *μ*m hole size. Mean radii of particles and standard deviations (excluding points with less than 1 per 1000 occurrences) are presented in Table 2.

As seen from Table 2 narrowest distribution is achieved in particles formed with spray caps of 4.0 *μ*m hole size. However this sample contains two types of particles; moreover, the bigger particles are above 3 *μ*m in diameter, which limits their applicability in an inhalation administration. A wide size distribution is observed for particles made using 5.5 *μ*m hole size, with a substantial contribution of sizes over 3 *μ*m. Microspherical particles formed using spray caps with 7.0 *μ*m hole size have relatively narrow size distribution. A large number of particles are less than 3 *μ*m in diameter, which is the optimal condition for inhalation delivery. Smaller deviation from the mean value for the spray caps with 7.0 *μ*m hole size compared to 5.5 *μ*m hole size spray caps was demonstrated earlier [39]. The disproportion between particle sizes and the hole size can be attributed to the interplay of solution viscosity and the surface tension during droplet formation [55, 56]. Bigger droplets formed using spray caps with 7.0 *μ*m hole size have less of a tendency for aggregation. The values of aerodynamic radii of the particles, calculated using equations 3–28 from [63], are presented in the Table 2. The aerodynamic diameters of the particles formed using spray caps with 7.0 *μ*m hole size are in the range between 1.3 and 2.9 *μ*m, which allow their application for inhalation delivery.

#### 3.5.6. Drug Content

PHE content in the composite determined using UV-spectrophotometry was 18.7 ± 1.8%, 17.0 ± 2.7%, and 18.0 ± 1.3% for microspherical particles, produced by spray drying using spray caps with 4.0, 5.5, and 7.0 *μ*m hole size, respectively.

The percent drug content in solid dispersions was found to be excellent and was approximately close to the theoretical values (16.7%). This proves that the spray drying method is suitable for the preparation of solid dispersions of PHE.

### 3.6. *In Vitro* Dissolution Study

Dissolution kinetics of the drug is one of the main factors considered for the development of the new drug delivery forms. Thereby the dissolution kinetics of PHE from the microspherical particles produced in the present study was investigated. Kinetic assays were performed at pH = 6.86, as it was earlier shown that pleural fluid of patients with some diseases has pH below 7 [64, 65].

**Figure 12:**
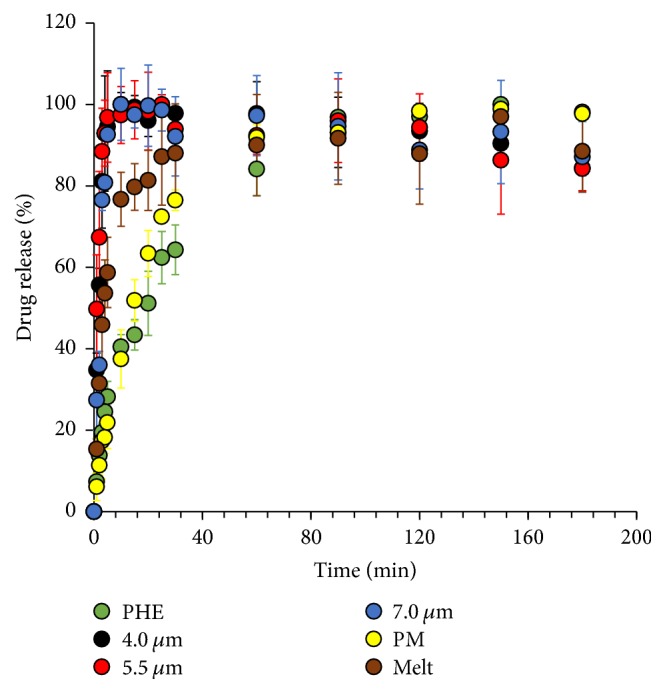
Dissolution profiles of crystalline PHE (green) and microspherical particles produced by spray drying using spray caps with 4.0 *μ*m (black), 5.5 *μ*m (red), and 7.0 *μ*m (blue) hole size, 1 : 5 physical mixture of PHE with PVP K29-32 (yellow), and 1 : 5 solid dispersion of PHE with PVP K29-32, formed by melting (brown) in a buffer solution, pH = 6.86.

As seen from kinetic curves presented in Figure 12 a polymer matrix increases the dissolution rate of PHE. Full dissolution of drug from the spherical particles is achieved in the first 10 minutes, while for pure PHE full dissolution was observed no earlier than after 90 minutes after the start of the experiment.

A study of dissolution kinetics shows that microspherical particles demonstrate fast dissolution rate, which permits their application in drug inhalation delivery systems. The rate of PHE dissolution is affected not only by the presence of polymer but also by the particle size.

## 4. Conclusion

Based on the DSC and PXRD data optimal ratio of PVP K29-32 : PHE was established to be 5 : 1 by weight. No crystal phase of PHE is formed, glass transition temperature of the polymer is well above spray drying condition, and the Gibbs energy of dissolution is negative for this composition. Based on the viscosity measurements of polyvinylpyrrolidone K29-32 solution in ethanol, an optimal concentration of the former in the latter was found to be 2.5% w/v.

Microspherical particles of solid dispersions of polyvinylpyrrolidone K29-32 with model hydrophobic drug, PHE produced using spray drying, were studied using several physicochemical methods: SEM, DSC, PXRD, and FTIR. It is found that the spray drying process parameters enable producing particles with relatively low solvent content, on which PHE stays in amorphous form for no less than 4 months. Particles prepared using spray caps with 7.0 *μ*m hole size have optimal size distribution for inhalation administration. The drug content in microparticles was found to be excellent and was approximately close to the theoretical values.

Measured dissolution kinetics of PHE evidence that dissolution rate of PHE from microspherical particles of solid dispersions allows their application as inhalation delivery systems with fast maximum concentration achievement time.

The results of the present study can be applied for the optimization of the spray drying process for the production of solid dispersion based on poorly soluble drugs with possible inhalation administration and enhanced bioavailability.

## Figures and Tables

**Table 1:** Values of Gibbs free energy of dissolution (Δ_*S*_*G*) of PHE in water at different ratios of PVP K29-32 : PHE.

PVP : PHE ratio	Δ_*S*_*G*, J/mol
1 : 1	4757 ± 301
2 : 1	2651 ± 200
4 : 1	1277 ± 67
5 : 1	−281 ± 34
6 : 1	−806 ± 110
8 : 1	−1991 ± 150
10 : 1	−3413 ± 446

**Table 2:** Values of maxima (curve peaks) and corresponding standard deviations of the microparticles radius presented in Figure 11. Calculated aerodynamic radii are shown in brackets.

Spray caps hole size	Peak (*µ*m)	Standard deviations (*µ*m)
4.0	0.42 (0.48)	0.31
	1.60 (1.82)	0.35
5.5	1.04 (1.19)	1.01
7.0	0.61 (0.70)	0.64
